# The sigma-1 receptor: a regulator of cancer cell electrical plasticity?

**DOI:** 10.3389/fphys.2013.00175

**Published:** 2013-07-16

**Authors:** David Crottès, Hélène Guizouarn, Patrick Martin, Franck Borgese, Olivier Soriani

**Affiliations:** ^1^Université de Nice, UMR 7277Nice, France; ^2^Institut de Biologie de Valrose, CNRS UMR 7277, INSERM U1091, Université de NiceNice, France

**Keywords:** sigma-1 receptor, chaperones, ion channels modulation, stress, physiological, cancer

## Abstract

Originally mistaken as an opioid receptor, the sigma-1 receptor (Sig1R) is a ubiquitous membrane protein that has been involved in many cellular processes. While the precise function of Sig1R has long remained mysterious, recent studies have shed light on its role and the molecular mechanisms triggered. Sig1R is in fact a stress-activated chaperone mainly associated with the ER-mitochondria interface that can regulate cell survival through the control of calcium homeostasis. Sig1R functionally regulates ion channels belonging to various molecular families and it has thus been involved in neuronal plasticity and central nervous system diseases. Interestingly, Sig1R is frequently expressed in tumors but its function in cancer has not been yet clarified. In this review, we discuss the current understanding of Sig1R. We suggest herein that Sig1R shapes cancer cell electrical signature upon environmental conditions. Thus, Sig1R may be used as a novel therapeutic target to specifically abrogate pro-invasive functions of ion channels in cancer tissue.

## Introduction

**Figure 1:**
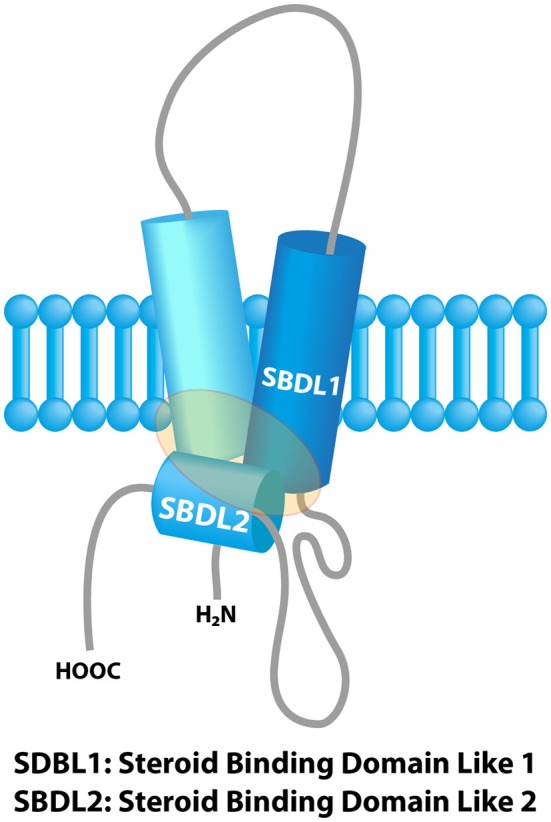
**Model of the sigma-1 receptor binding region from previous photolabeling studies**. The shaded area represents the ligand binding region. Adapted with permission from Chu et al. (2013).

The concept of “Sigma receptors” arose 40 years ago in a pharmacological study postulating the existence of three types of opioid receptors on the basis of the psychomimetic effects induced by several opioid compounds (μ, κ and σ receptor respectively accounting for the effects produced by morphine, ketacyclazocine and SKF 10,047) (Martin et al., 1976). Further pharmacological studies revealed the existence of two binding sites, namely Sigma 1 (Sig1R) and Sigma 2 receptors (Sig2R) (Quirion et al., 1992). The Sig1R was cloned in 1996 (gene names: SIGMAR1 or OPRS1) and the gene is located on 9p13 (Hanner et al., 1996; Prasad et al., 1998). Sig1R is a 25-kDa protein anchored in the endoplasmic reticulum (ER) with no similarity with other known mammalian proteins, thus definitely ruling out any connection with a classical receptor family. Sig1R possesses two transmembrane regions and two steroid binding domains (SBD). These domains form a pocket which is the binding site for cholesterol, steroids, sphingolipids (Palmer et al., 2007; Fontanilla et al., 2008), and also for a wide panel of synthetic or natural compounds (sigma ligands) from different classes such as opioids, antipsychotics, psychostimulants, alkaloids or antidepressants (Pal et al., 2008; Maurice and Su, 2009) (Figure 1). *In vivo*, endogenous dimethyl tryptamine (DMT) interacts with Sig1R in the brain but its physiological significance as an endogenous sigma ligand is not yet clarified (Fontanilla et al., 2009; Mavlyutov et al., 2012).

The molecular nature of Sig2R has long been questioned. A recent work proposed the progesterone receptor membrane component 1 (Pgrmc1) as the putative sigma 2 binding site (Xu et al., 2011). This cytochrome-related protein binds several P450 proteins and various chemical compounds and it participates to cholesterol synthesis. However, while this putative Sig2R shares some pharmacological properties with Sig1R, the two proteins belong to distinct families. This review will focus on Sig1R.

Sig1Rs have been associated with many diseases including stroke, cocaine addiction, Alzheimer's disease, amnesia, amyotrophic lateral sclerosis, retinal degeneration, and cancer (Romieu et al., 2004; Aydar et al., 2006; Renaudo et al., 2007; Maurice and Su, 2009; Luty et al., 2010; Mavlyutov et al., 2011; Ruscher et al., 2011; Kourrich et al., 2013). Nonetheless, the way Sig1R operates in such diseases is still poorly understood. Su and colleagues'work on neurons and CHO cells have shed light on the molecular mechanisms underlying Sig1R functions. Sig1R is mainly located at the ER, in close contact with the mitochondria, in the so-called mitochondria-associated-ER membrane domains (MAM). In resting condition, Sig1R resides in ceramide- and cholesterol-rich lipid microdomains associated with the ER-resident chaperone GRP78 (BiP) (Hayashi and Su, 2007; Hayashi and Fujimoto, 2010). Under cellular stress leading to ER injury, Sig1R dissociates from BiP and binds IP_3_ receptors, enhancing in turn cell survival through the control of calcium signaling between the ER and mitochondria. In addition, Sig1R translocates to other cell compartments and binds to different membrane proteins. The stimulation with sigma “agonists” mimicks stress-induced Sig1R dissociation from BiP and Sig1R delocalization, while sigma ligands classified as “antagonists” impede this process (Hayashi and Su, 2007). Altogether, these results have led to a model in which Sig1R is “silent” in normal physiological conditions, whereas in case of a disease, Sig1R behaves as a chaperone that binds client protein to the benefit of cell survival (Su et al., 2010). This exciting hypothesis has been validated by recent studies demonstrating that Sig1R molecular silencing reduces brain recovery after experimental stroke (Ruscher et al., 2011) and promotes retina degeneration after acute damage to the optic nerve (Mavlyutov et al., 2011).

The question of client proteins targeted by Sig1R is of importance. Beyond the coupling with IP3 receptors, a number of studies mainly based on the effects of exogenous sigma ligands have shown that Sig1R interferes with dopamine and acetylcholine systems and modulates the function of ion channels belonging to various families. Recent studies have described a molecular interaction between Sig1R and ion channels, suggesting that ion channels represent a major client protein family for the Sig1R chaperone (Carnally et al., 2010; Crottes et al., 2011; Balasuriya et al., 2012; Kourrich et al., 2012). Over the past decades, ion channels have been integrated to the main cellular processes underlying the hallmarks of cancer: tumors often express ion channels and transporters that are absent from the corresponding tissue. It is suggested that these channels and transporters enhance the cell's capacity to adapt themselves to restraint metabolic conditions encountered within the tumor tissue (low pH and PO_2_, poor nutrient supply, etc… ) (Wulff et al., 2009; Prevarskaya et al., 2010; Arcangeli, 2011). Transport proteins therefore participate to the adaptive cancer cells'response to environmental stress, conferring them with greater aggressiveness. This review attempts to draw together the knowledge about ion channel regulation by Sig1R and the recent discoveries on the function of ion channels in cancer. We suggest that upon environmental challenging conditions within the tumor, Sig1R may participate in the electrical remodeling of cancer cell electrical properties to enhance their survival and aggressiveness.

## Sigma 1 receptors in cancer

Binding experiment studies realized in the 90's revealed that sigma receptors are highly expressed in many human and rodent tumor cell lines including breast, lung, prostate, colon, melanoma, neuroblastoma and glioma (John et al., 1995; Vilner et al., 1995b; Aydar et al., 2004). However, most of the sigma ligands used in these studies are not selective enough between Sig1R and Sig2R to draw a definitive conclusion on the density of each binding site in the explored cancer cell types. Using a specific Sig1R antibody, a high expression of Sig1R was found in lung, breast and prostate cancer cell lines whereas low levels were found in normal counterpart cells. Interestingly, the Sig1R density was increased in high metastatic potential cancer cells suggesting a link between Sig1R expression and aggressiveness (Aydar et al., 2006). In another study, the expression of Sig1R was explored by imunohistochemistry in 58 breast cancer patients and 51 normal breasts. Sig1R positive epithelial cell staining was detected in 60 or 41% of invasive or *in situ* cancers respectively, in 75% of ductal hyperplasia and in 33% of normal breast (Wang et al., 2004). Accordingly, scintigraphy with a moderately-selective Sig1R ligand (*N*-[2-(1′-Piperidinyl) Ethyl]-3-^123^I-Iodo-4-Methoxybenzamide) on patients with primary breast cancer revealed that the ligand was specifically retained within the tumor site, but not in healthy tissues (Caveliers et al., 2001). Several reports indicate that the use of Sig1R ligands to target therapeutic nanoparticles dramatically enhances the delivery of siRNA or drugs at the tumor site in melanoma, prostate, lung and breast cancer (Li and Huang, 2006; Chen et al., 2010; Guo et al., 2012; Kim and Huang, 2012).

Altogether, these studies strongly suggest that Sig1R is over expressed in many cancer cells and an extensive exploration of Sig1R expression in biopsies from various cancers is now required to determine whether Sig1R could be proposed as a diagnosis or prognosis marker.

The effects of sigma ligands on cancer cells' behavior have been assessed by many groups *in vitro* and *in vivo*. Early descriptive works showed that cell treatment with sigma ligands causes rounding, detachment and growth inhibition of C6 glioma (Vilner et al., 1995a), breast and colon carcinoma and melanoma cells (Brent and Pang, 1995; Aydar et al., 2004). Further works by Spruce and coll. showed that the moderately selective ligand rimcazole provokes *in vitro* and *in vivo* (mouse xenograft model) a tumor-selective, caspase-dependent apoptosis of breast and colon cancer (Spruce et al., 2004; Achison et al., 2007). Rimcazole was shown to antagonize a Sig1R-dependent mechanism involving a calcium-dependent activation of phospholipase C, a calcium-independent inhibition of phosphatidylinositol 3′-kinase pathway signaling and the accumulation of HIF-1α. While Sig1R agonists (+) pentazocine and (+) SKF10,047 had no effect *per se*, these ligands could abrogate rimacazole-induced apoptosis, suggesting that in cancer cells, Sig1R is in an activated state and enhances survival. In agreement with this hypothesis, transfection of Sig1R in HEK293 cells reverses apoptosis induced by the over-expression of Bax or by staurospaurine (Spruce et al., 2004; Achison et al., 2007; Crottes et al., 2011). However, whether Sig1R protects cancer cells from death through a chaperoning activity has not yet been addressed.

Sig1R has also been connected to cell/matrix interaction. Aydar et al. have demonstrated that in breast cancer cells, Sig1R is associated with β1 integrin in lipid cholesterol-enriched rafts. Silencing Sig1R with siRNA chased β1 integrin from lipid rafts, reducing cell adhesion to matrix component such as fibronectin and vitronectin. Interestingly, treatment with the Sig1R agonist SKF10,047 also reduced β1 integrin density within lipid rafts and cell adhesion, an effect that was mimicked by the depletion of membrane cholesterol by methyl-β-cyclodextrin (Palmer et al., 2007).

From these data, it is clear that Sig1R participates on several facets of cancer cell biology. Recently, mutations in Sig1R have been found to cause a form of ALS and frontotemporal lobar degeneration (Luty et al., 2010; Al-Saif et al., 2011; Prause et al., 2013). Whether mutations in Sig1R also occur in cancer tissues is a question that remains to be explored. So far, the common mechanism by which Sig1R or sigma ligands drives cancer cell behavior is not clear. An exciting hypothesis arises from converging studies describing Sig1R as a sterol-dependent, stress-activated chaperone controlling lipid raft formation in the ER and the plasma membrane (PM) [extensively reviewed in Tsai et al. (2009), Hayashi and Su (2010)]. In response to environmental conditions encountered in cancer tissue (hypoxia, nutrient and growth factor deprivation) Sig1R may dynamically trigger various adaptation mechanisms, the nature of which being tightly dependent on the client protein available in a given tumor cell type. At this stage, it is noteworthy that ion channels emerge from the literature as the main client protein family for Sig1R (Hayashi and Su, 2007; Crottes et al., 2011; Balasuriya et al., 2012; Kourrich et al., 2012, 2013).

## Sig1R: a modulator of ion channels

### Voltage-gated ion channels

Voltage-gated ion channels (VGIC) are mainly involved in the initiation and shaping of action potentials and global cell excitability (Hodgkin and Huxley, 1952; Hille, 1984). The progress made during the past decade in characterizing the electrical signature of cancer cell has intriguingly extended the initial function of VGIC far beyond the field of exciting cells. Indeed, VGIC are involved in a number of tumor cell processes including mitosis (Weber et al., 2006; Becchetti, 2011), migration (Gillet et al., 2009; Becchetti and Arcangeli, 2010), apoptosis (Lang et al., 2004), adhesion to ECM (Pillozzi and Arcangeli, 2010), angiogenesis (Pillozzi et al., 2007), homing and drug resistance (Pillozzi and Arcangeli, 2010). Interestingly, Sig1R has been shown to interact with K^+^, Ca^2+^, Cl^−^ and Na^+^ channels (Renaudo et al., 2004; Kourrich et al., 2012). Very recent studies have provided some clues about these interactions.

#### 1- voltage-gated K+ channels (VGKC)

Numerous studies have reported the inhibition of VGKC by sigma ligands in a wide range of cell types (Kennedy and Henderson, 1990; Soriani et al., 1998, 1999a,b; Lupardus et al., 2000; Kourrich et al., 2012). In particular, sigma ligands decrease current density and provoke a leftward shift in the voltage-dependency inactivation (Zera et al., 1996; Soriani et al., 1999a; Aydar et al., 2002). In a study performed in frogs'pituitary cells, it was nonetheless shown that sigma ligands depress the M-current by a rightward shift of the activation curve (Soriani et al., 1999b). The mechanism by which sigma ligands modulate Kv channels has been proposed to be either direct or indirect, depending on the model used. Inside-out patch clamp experiments suggested a direct effect of sigma ligands on Kv channels in rodent neurohypophysal terminals and in small cell lung carcinoma (Wilke et al., 1999; Lupardus et al., 2000). However, in frogs'pituitary cells, the inhibitory effects of the selective Sig1R ligand (+) pentazocine, on both delayed-rectifier and I_A_ currents, were abolished in the presence of cholera toxin, GTPγS or GDPβS suggesting the involvement of a Gs-protein dependent pathway (Soriani et al., 1998, 1999a). How could these two sets of observation be interpreted? In a recent study, Mei et al. showed that the sigma ligand Cyproheptadine stimulates the Kv2.1-dependent current in cortical neurons in a Sig1R and G_i/o_-dependent manner. The study indeed describes a functional interaction between Sig1R, Kv2.1 and a G-protein coupled receptor (GPCR): the mu-opoid receptor (He et al., 2012). It can then been proposed that sigma ligands, either alter directly the Sig1R/VGKC coupling or modulate functional complexes that integrate Sig1R, VGKC and GPCR. This last hypothesis is strengthened by recent reports demonstrating that Sig1R modulates several GPCR in the brain (i.e. opioid and muscarinic acetyl choline receptors) (Kim et al., 2010), and forms a complex with D1 and D2 dopamine receptors (Navarro et al., 2010, 2013).

Whether Sig1R requires an endogenous/exogenous sigma ligand to modulate VGKC is a crucial question that has been addressed in a few but important reports focusing on the molecular interaction between Sig1R and its partners. In *Xenopus oocytes*, it has been shown that the co-expression of Sig1R with Kv1.4 or Kv1.3 accelerates the inactivation kinetic parameters (Aydar et al., 2002; Kinoshita et al., 2012). In human leukaemic cells, our group found that the silencing of Sig1R by shRNA reduces the endogenous human ether-à-gogo-related gene (hERG; Kv11.1) current density without altering channel voltage dependency or kinetic parameters. Delving into the molecular mechanisms, we observed that the silencing of Sig1R decreases hERG maturation efficiency and diminishes the α-subunit channel stability at the plasma membrane, in turn reducing the number of ion channels available (Crottes et al., 2011). Inasmuch Sig1R co-immunoprecipitates with hERG, these observations are consistent with the idea of a Sig1R protein behaving either like a chaperone or a channel regulatory β-subunit through a protein/protein interaction (Aydar et al., 2002; Crottes et al., 2011; Kinoshita et al., 2012). This hypothesis was further strengthened by a recent report showing that cocaine exposure induces in nucleus accumbens a persistent protein/protein association between Sig-1Rs and Kv1.2 channels. This phenomenon is associated with a redistribution of both proteins from the intracellular compartments to the plasma membrane (Kourrich et al., 2013).

The presence of VGKC is linked to cell proliferation in various cancer types, through the regulation of both the resting (controlling the Ca^2+^ driving force) and cell volume, both phenomenon participating in the cell cycle checkpoints (for review; Wulff et al., 2009; Becchetti, 2011; Felipe et al., 2012). The connection between VGKC, Sig1R and cancer cell proliferation has been first addressed by Renaudo et al. (2004). We observed that selective Sig1R ligands provoke a cell cycle arrest in small cell lung carcinoma and T-ALL cells by blocking the delayed-rectifier and Kv1.3 channels, respectively. In both cases, Sig1R-dependent inhibition of potassium currents resulted in an accumulation of the cyclin inhibitor p27^Kip1^ and a reduction in cyclin A contents, leading to an arrest at the end of the G_1_ phase of the cell cycle (Renaudo et al., 2004).

VGKC of the ether-à-gogo family, i.e., hERG and EAG channels, represent a source of highly promising therapeutic targets for cancer. hERG is mainly expressed in the heart, the central nervous system and the endocrine system were it regulates the frequency of action potentials (for review: Vandenberg et al., 2012). In a series of excellent papers, Arcangeli's group has demonstrated that hERG is a tumor marker of myeloid and lymphoid leukaemias, colon and breast carcinoma, ovarian cancer and glioblastoma (Pillozzi et al., 2007, 2011; Pillozzi and Arcangeli, 2010; Arcangeli, 2011). Importantly, they demonstrated that upon β1 integrin stimulation, hERG forms signaling macro-complexes with β1-integrin, the VEGF receptor Flt-1 or the cytokine receptor CXCR4 in lipid rafts. The channel in turn participates in a crosstalk between cancer cells and their microenvironment to promote invasive processes such as motility, angiogenesis, homing and chemoresistance (Pillozzi et al., 2007; Pillozzi and Arcangeli, 2010). As stated above, we recently showed that Sig1R expression stimulates hERG maturation and membrane stability in the chronic myeloid leukaemia cell line K562. Inasmuch Sig1R co-immunoprecipitates with both immature and mature forms of the channel α-subunits, it is suggested that Sig1R not only associates with hERG in the ER, but also drives it to the plasma membrane (Crottes et al., 2011). The question of whether Sig1R is involved in the formation of such hERG-dependent signaling complexes with β-integrins and other partners is an interesting one but has not been addressed yet. This hypothesis deserves further consideration *in vitro* and *in vivo* knowing that both Sig1R silencing and treatment with the sigma ligand igmesine decrease K562 cell adhesion capacity to fibronectin in a hERG-dependent manner (Crottes et al., 2011).

Channels of the EAG family are present in a number of tumor types. Stuhmer's group nicely demonstrated that CHO cells transfected with EAG exhibit a cancerous invasive phenotype *in vitro* and *in vivo* (Hemmerlein et al., 2006; Gomez-Varela et al., 2007; Pardo and Suhmer, 2008). Further signaling studies revealed that EAG-1 (Kv10.1) enhances cell resistance to hypoxia by increasing HIF-1 levels, thus stimulating VEGF secretion (Downie et al., 2008). The over expression of EAG-2 has been recently shown in human medulloblastoma (MB). In this cancer, EAG-2 promotes the progression of the MB tumor by regulating cell volume dynamics, in turn inhibiting the tumor suppressor p38 MAPK pathway (Huang et al., 2012). While the putative link between EAG channels and Sig1R has not been addressed so far, it is tempting to speculate such an interaction considering the molecular and structural proximity between EAG and hERG channels.

#### 2-voltage-gated Na^+^ channels (VGNC)

The existence of VGNC has been first speculated by Hodgkin and Huxley to account for the fast depolarizing phase of the action potential of excitable cells (Hodgkin and Huxley, 1952). To date, the family of VGNC includes nine members mainly involved in the encoding of neuronal signaling, cardiac rhythm, muscle contraction and endocrine secretion (Hille, 1984; Harmar et al., 2009). Intriguingly, VGNC are expressed in metastatic cells of many cancers. In these cells, the sodium current driven by VGNC α subunits enhances the invasion and metastasis *in vivo* (Brisson et al., 2011, 2012; Yang et al., 2012). Expression of the cardiac Nav1.5 α subunit (SCN5A) is correlated with a poor prognosis in breast cancer specimens, suggesting that VGSCs may be used as prognosis marker in cancer progression (House et al., 2010; Yang et al., 2012). The mechanical link between Nav1.5 and cancer progression has been recently documented: in breast cancer cells, Nav1.5 associates with the Na^+^/H^+^ exchanger NHE1 in caveolae; Nav1.5 stimulates NHE1 activity, contributing to the acidification of the pericellular space. The low extracellular pH in turn potentiates the activity of different cathepsins involved in ECM degradation, a fundamental step for cancer cell invasion process (Gillet et al., 2009; Brisson et al., 2011, 2012). In recent studies, Jackson and Ruoho's groups have shown that sigma ligands reduce Nav1.5-dependent currents in cardiomyocytes of wild type mice. Interestingly, Nav1.5 current sensitivity to sigma ligands was lost in cardiomyocytes of knock-out mice for Sig1R (Fontanilla et al., 2009; Johannessen et al., 2011). The nature of the interaction occurring between Sig1R and Nav1.5 has been scrutinized in 2012 by atomic force microscopy (AFM) which revealed that Sig1R directly binds the channel with a four-fold symmetry in human embryonic kidney cell (HEK) heterologous expression system (Balasuriya et al., 2012). Because the Nav1.5 channel includes the four pore-forming α subunits within a single protein, this result suggests that Sig1R neither interacts with C- nor N-terminus, but rather with the transmembrane domains. This hypothesis is strengthened by the fact that deletions in the transmembrane domain of Kv1.3 subunits abolish their co-immunoprecipitation with Sig1R in *Xenopus* oocytes (Kinoshita et al., 2012). While the suppression of Sig1R expression in mice cardiomyocytes fails to alter any parameter of the native Nav1.5 current (Fontanilla et al., 2009), the Sig1R silencing in the highly aggressive MDA-MB-231 breast cancer cell line results in a strong reduction in current density, suggesting that Sig1R controls Nav1.5 trafficking in cancer cells but not in healthy cardiac cells (Balasuriya et al., 2012). From these observations, it can be hypothesized that Sig1R, by enhancing Nav1.5 membrane expression in breast cancer cells, modulates NHE1 activity, resulting in greater aggressiveness potency.

#### 3-voltage-gated calcium channels (VGCC)

VGCC are principally involved in fast synaptic transmission, cardiomyocyte and striated muscle contraction, as well as stimulus-secretion coupling. The low threshold T-type channel (Cav-3) has however been involved in proliferation and differentiation in several cancer cell lines (Gackiere et al., 2008; Prevarskaya et al., 2010; Becchetti, 2011). While no data support an interaction between Sig1R and the T-type channel in the literature, it has been shown that Sig1R co-immunoprecipitate with high-threshold L-type channels in retinal ganglion and that the sigma ligand SKF 10.047 inhibits the corresponding current (Tchedre et al., 2008). These observations suggest that VGCC in cancer cells might be a client for Sig1R.

### Calcium-activated potassium channels (KCa)

KCa channels are involved in many physiological processes by regulating calcium entry through the control of the membranes'resting potential and Ca^2+^ driving force. A link between Sig1R and small-conductance KCa channels has been recently proposed in synaptic activity and plasticity in the hippocampus (Martina et al., 2007). In this report, the authors showed that Sig1R ligands potentiate the N-Methyl-D-Aspartate (NMDA) receptor responses and long-term potentiation (LTP) by inhibiting a small conductance Ca^2+^-activated K^+^ current (SK channel). Interestingly, SK3 channels play a predominant role in melanoma and breast cancer cell migration and are considered as potent targets for cancer therapy (Potier et al., 2006; Chantome et al., 2009; Girault et al., 2012). On the other hand, SK4 channels have been involved in the migration potency of glioblastoma stem cells (Ruggieri et al., 2012). The putative interaction between SK channels and Sig1R thus constitutes an interesting hypothesis that remains to be explored.

### Volume-regulated chloride channels (VRCC)

VRCC, functionally coupled with K^+^ channels, drive cell volume regulation by controlling chloride salt-associated water efflux (Hoffmann et al., 2009). Cell volume regulation and regulatory volume decrease (RVD) participate to at least three main aspects of cancer progression, i.e., cell cycle (G1/S and G2/M volume checkpoints) (Lang et al., 2000; Rouzaire-Dubois et al., 2000; Becchetti, 2011; Hoffmann, 2011; Huang et al., 2012), motility (control of cell shape dynamics through salt and water fluxes) (Cuddapah and Sontheimer, 2011) and apoptosis (Apoptosis Volume Decrease occurring at an early signaling step of programmed cell death) (Bortner and Cidlowski, 2011; Bortner et al., 2012). Our group has shown that sigma ligands strongly inhibit both VRCC and VGKC in T leukaemic and small cell lung carcinoma cells in a Sig1R-dependent manner. VRCC and VGKC inhibition lead to a strong reduction in RVD potency after a hypotonic shock. In isotonic conditions, cell treatment with sigma ligands lead to cell swelling, underlying an arrest of cell division at the late G_1_ phase. These results indicate that the pharmacological alteration of Sig1R, by inhibiting channels involved in RVD, can block the cell division process (Renaudo et al., 2004, 2007). In these studies we also questioned the function of Sig1R in cancer cells in the absence of exogenous ligands. We observed that the over-expression of Sig1R in HEK cells was sufficient *per se* to significantly reduce the activation kinetics of VRCC upon hypotonic shock. We proposed that the presence of Sig1R induces a tonic reduction of VRCC activity, not sufficient to impede the cell cycle, but strong enough to protect cells from apoptosis by delaying AVD. This result was confirmed by showing that cells over-expressing Sig1R are less sensitive to staurospaurine-induced apoptosis than normal cells (Renaudo et al., 2007). Together with other reports, this study has unveiled the function of Sig1R as a protein involved in cell protection against environmental stress by modulating ion channels (Hayashi and Su, 2007; Renaudo et al., 2007).

### Calcium signaling and ion channels at the MAM

Cell fate largely depends on calcium exchanges occurring between ER and mitochondria. These exchanges generally take place at specific membrane localization, the MAMs, which were originally described as sites for lipid synthesis and lipid transfer between ER and mitochondria membranes (Rusinol et al., 1994) for review: (Parys et al., 2012). Calcium fluxes between the two compartments involve various chaperones and signaling proteins as well as ion channels and transporters including IP3 receptors, voltage-dependent anion channels (VDAC) or the translocon. The regulation of calcium entry in the mitochondria participates in the control of the energy state and cell response to ER-mediated stress. Calcium homeostasis at MAM therefore constitutes a crossroad decision for cell engagement toward apoptosis, survival or autophagy (Tsai et al., 2009; Parys et al., 2012; Hammadi et al., 2013). Not surprisingly, MAM-associated transport machinery is disregulated in the context of environmental challenges in cancer such as hypoxia, low pH and dramatic nutrient deprivation (Moenner et al., 2007; Raturi and Simmen, 2013). As stated above, Hayashi and Su have demonstrated in CHO cells that the Sig1R chaperone plays a fundamental role in regulating the Ca^2+^ transport machinery within the MAMs, leading to a reinforced cell survival in response to environmental stress (Hayashi and Su, 2007). In particular, stress-activated Sig1R chaperones IP3 receptor and prevents its degradation. Moreover a recent report has shown that Sig1R is physically associated to VDAC2, a mitochondrial channel involved in cholesterol import into the mitochodria for metabolic regulation (Marriott et al., 2012). While no data is available on the function of MAM-associated Sig1R in tumors, it is conceivable that Sig1R contributes to the adaptation of cancer cells in restrictive environment.

## Conclusion and perspectives

**Figure 2:**
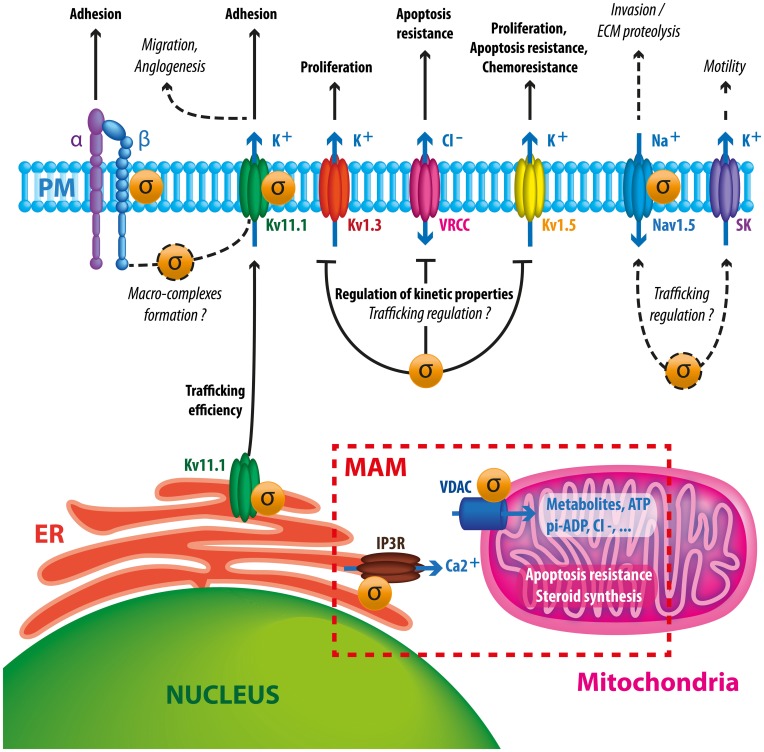
**Schematic diagram summarizing putative and documented interactions between Sig1R and ion channels in cancer cells**. Sig1R promotes hERG maturation and membrane stability which may potentiate channel interaction with β1 integrin macrocomplexes, leading to enhanced migration and angiogenesis. Sig1R also directly binds to other VGIC such as Kv1.3, Kv1.5, and Nav1.5 controlling either current density or kinetic properties of channels. These interactions may have consequences on cell proliferation, apoptosis resistance, chemoresistance and invasive properties. The presence of Sig1R in cancer cells tonically reduces VRCC activity via a yet unknown mechanism, leading to a better resistance to apoptotic signals. Sigma receptors have also been associated to SK channels, suggesting that they may participate to cancer cell motility. At the MAM, Sig1R may enhance cell survival by regulating calcium fluxes between ER and mitochondria by chaperoning IP3 receptors and VDAC channels.

In summary, Sig1R is a stress-activated chaperone which controls, through different mechanisms, several families of ion channels at the plasma membrane and at the MAM. Studies realized in the retina, brain and heart strongly suggest that Sig1R participates in cell resistance to tissue injury, for instance infarction, stroke or ischemia (Kourrich et al., 2012). Several reports indicate that Sig1R exerts a role only in conditions of stress and remains generally “silent” in healthy organs or in steady-state conditions (Maurice and Su, 2009; Tsai et al., 2009). In good agreement with this idea, Sig1R KO mice present a normal development and behavior but are less resistant to experimental stroke (Ruscher et al., 2011). Moreover, the absence of side effects of Sig1R ligands in clinical trials in human suffering psychiatric disorders, improves the hypothesis of a dynamic and protective role of Sig1R in stressing conditions (Volz and Stoll, 2004; Banister and Kassiou, 2012). Thus, it is tempting to speculate that tumor cells hijack the primary protective function of Sig1R to enhance their survival/growing/invasive potency in restrictive metabolic conditions encountered within the tumor tissue. As demonstrated by many authors, the aberrant expression of ion channels confers selective advantages for cancer cells to adapt their behavior and survival in the tumor environment. While research studies mainly focus on the function of one ion channel in a cancer type, it is important to consider that many ion channels are deregulated in the same cancer cell. Because a variety of ion channels are client proteins for Sig1R, we speculate that the Sig1R chaperone controls cancer cells'electrical plasticity by putatively “driving” ion channels to potentiate their function in proliferation, apoptosis resistance, migration and angiogenesis (Figure 2). At the time being, there is no real explanation on the process that controls the expression of all these ion channels in cancer cells and it is often postulated that this is due to the acquisition of an embryonic or developmental phenotype. The possibility that Sig1R expression might participate to this phenotype is an interesting hypothesis that has not been explored so far.

The literature strongly argues for a close interaction between Sig1R and ion channels that are already expressed in the cell. An alternative mechanism should however be considered: because of the spatial dynamics of Sig1R within the cell, the protein could also behave as a transcriptional factor controlling either directly or indirectly a kit of genes encoding ion channels. While no data supports the presence of Sig1R in the nucleus, many reports have shown the involvement of Sig1R in a number of signaling pathways potentially targeting transcriptional activity (i.e., MAP kinases, PKA, PI3K/AKT, NFk-B, c-Fos, CREB) (for review: Hayashi et al., 2011).

It is noteworthy that ion channels expressed in cancer cells play important functions in healthy organs as well such as in the heart and brain. As a consequence, therapies based on toxins and drugs directly targeting ion channels present major drawbacks for cancer treatment. The unique properties of Sig1R may pave a new avenue to alter ion channels specifically within tumors. In this regard, many outstanding questions need to be addressed to unravel the importance of Sig1R in cancer such as the consequences of Sig1R silencing on the electrical signature of cancer cells and subsequent alteration of their behavior *in vitro* and *in vivo*. Promising anti-tumoral effects have been obtained *in vivo* with exogenous sigma ligands, but the innate function of Sig1R in cancer remains undetermined. Moreover, the molecular mechanisms of Sig1R ligands on Sig1R/ion channel complexes remain to be addressed.

Answers to these questions will open new strategies based on the targeting of Sig1R to target ion channels and associated cancer progression.

### Conflict of interest statement

The authors declare that the research was conducted in the absence of any commercial or financial relationships that could be construed as a potential conflict of interest.
